# Supplementary material for: Autoimmune Rheumatic Disease Flares with Myocarditis Following COVID-19 mRNA Vaccination: A Case-Based Review
Source: Vaccines (Basel). 2022 Oct 21;10(10):1772. doi: 10.3390/vaccines10101772 (PMC9609433; doi:10.3390/vaccines10101772)
Supplement: Supplementary file 1 [file vaccines-10-01772-s001.zip › vaccines-1949251-supplementary.pdf]

Supplementary Materials

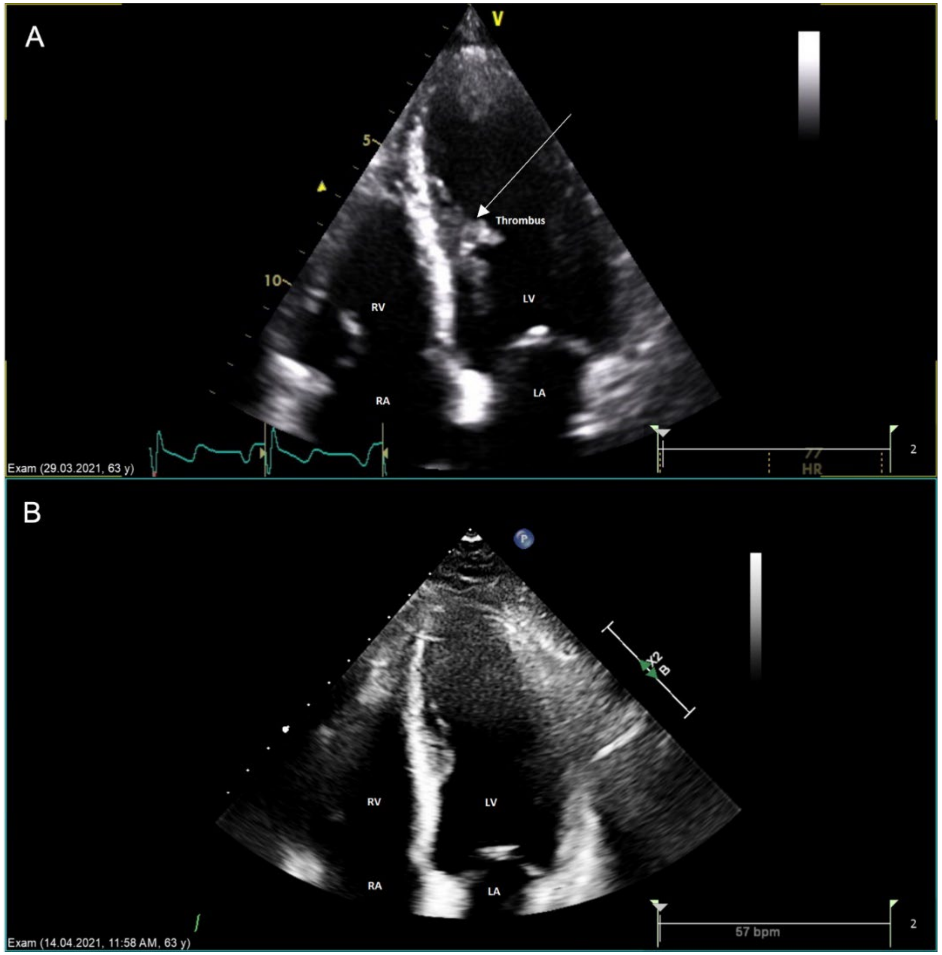

Figure S1. Trans-thoracic echocardiogram (TTE) four-chamber view in diastolic phase images of Case 2 showing (A) thrombi (white arrow) attached to the mid inferoseptal wall and (B) resolution of thrombi post treatment on repeat TTE 2 weeks later.

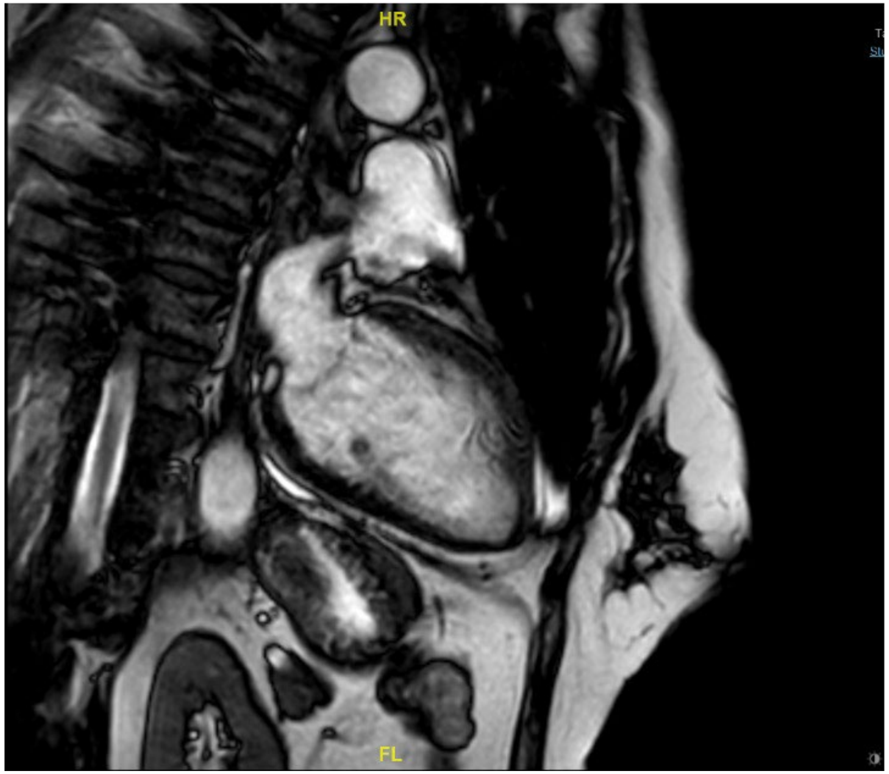

Figure S2. Steady-State Free Precession (SSFP) MRI sequence of Case 1 showing akinetic mid anterior wall in non ischemic pattern (white arrow) in diastolic (A) and systolic (B) phase.

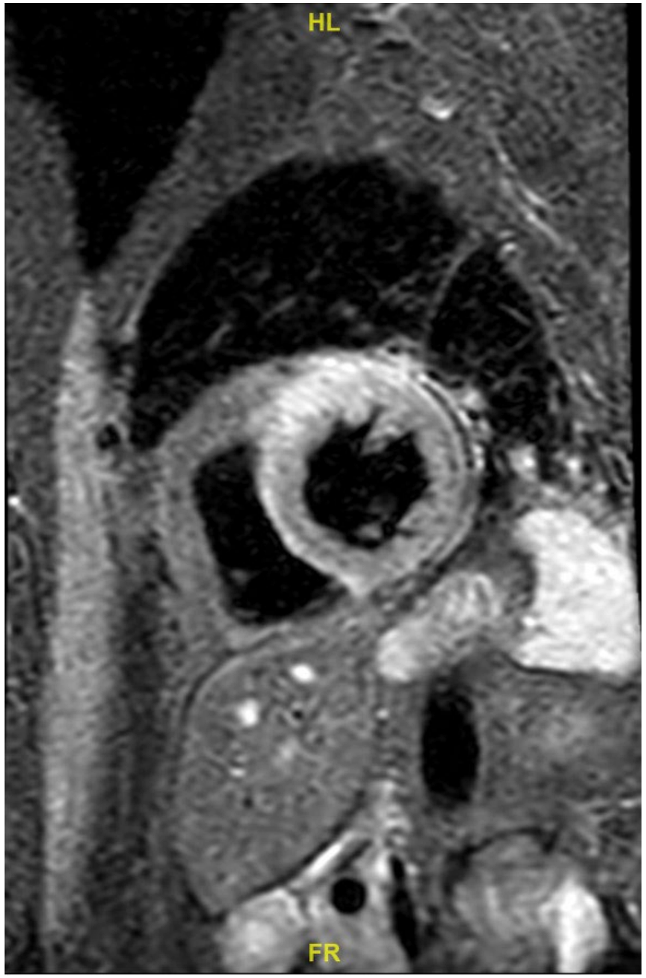

Figure S3. T2 weighted Short-Tau Inversion Recovery (T2w-STIR) sequence of Case 1 showing increased signal intensity in the mid-anterior wall segment demonstrating localized myocardial edema (white arrow).

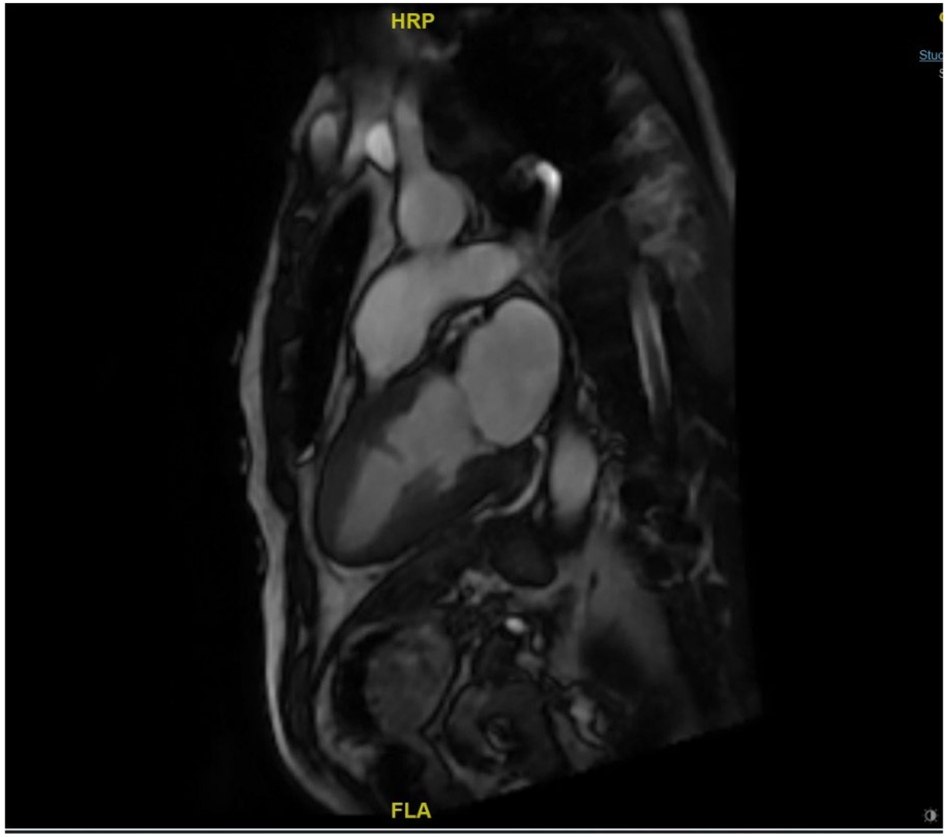

Figure S4. Steady-State Free Precession (SSFP) MRI sequence of Case 2 showing a mass (white arrow) attached to the endocardial surface of the mid-anterior segment of the left ventricle, suggestive of endomyocardial fibrosis with eosinophilic thrombi.

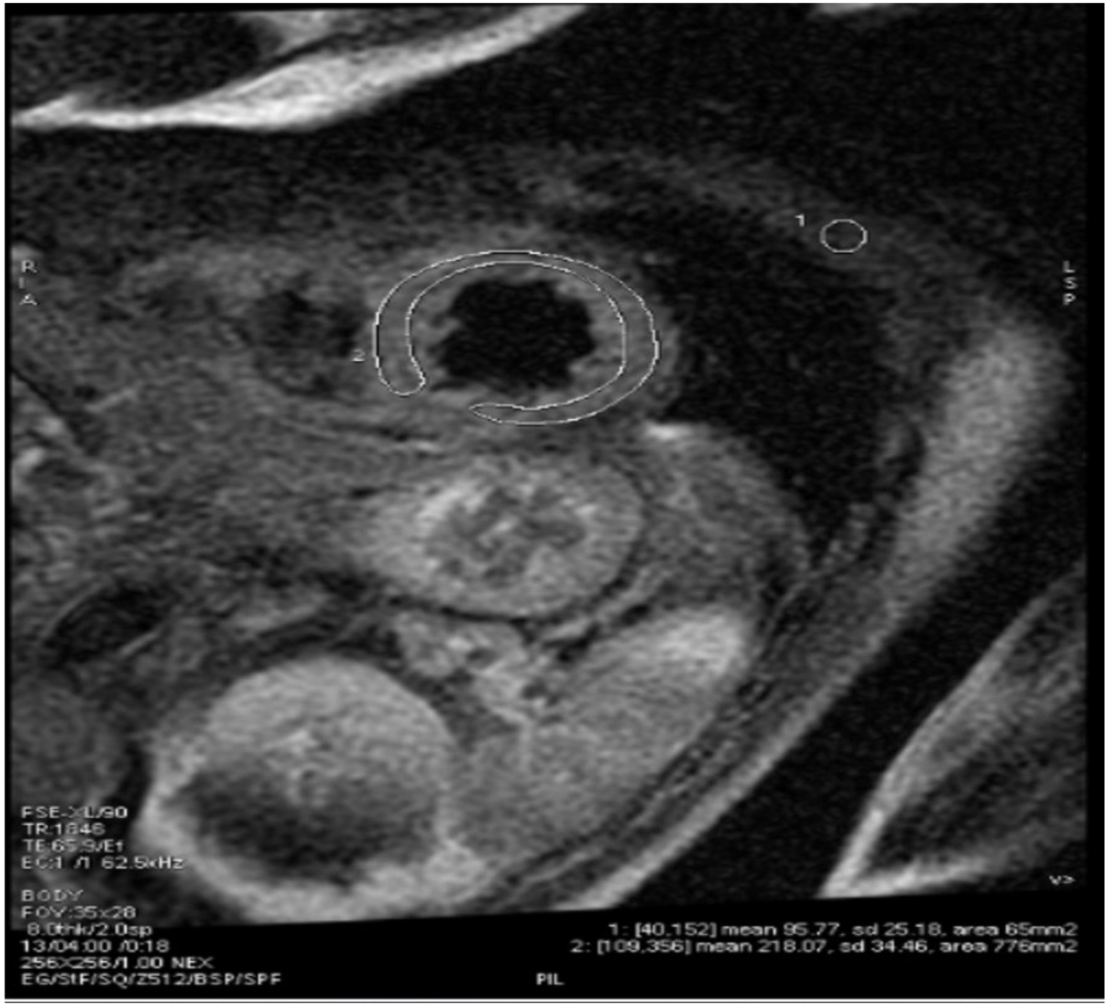

Figure S5. T2 weighted Short-Tau Inversion Recovery (T2w-STIR) sequence of a representative short axis left ventricular slice in Case 2, showing a Global STIR ratio of 2.3 (n:<1.9), demonstrating diffuse myocardial edema (white arrow).

Table S1. Demographics and medical background of patients.

	Patient 1	Patient 2	Patient 3	Patient 4	Patient 5
Demographics: Age (years) / ethnicity / gender	63 / Chinese / female	63 / Chinese / male	58 / Chinese / male	43 / Burmese / female	31/not reported/female
Medical and COVID-19 vaccination history					
ARD history *Diagnosis (manifestations)*	EGPA (left sciatic neuropathy, asthma, allergic rhinitis, peripheral eosinophilia, negative ANCA, positive anti-PR3)	Nil	SLE (anemia, thrombocytopenia, hypocomplementaemia, secondary Sjögren's)	SLE (Evans syndrome, Class 1/2 lupus nephritis with proteinuria, hypocomplementaemia, positive anti-dsDNA)	SLE with secondary APLS (Inflammatory arthritis, livedo reticularis, hypocomplementemia, positive APAs, ANA 1:1280 speckled, anti-Ro/Ro52/La, anti-dsDNA)
Treatment	AZA 50mg OM	N.A.	Pred 8-9mg/day and HCQ 200mg/day; defaulted treatment	AZA 150mg/day, HCQ ~285mg/day and Pred 6mg/day (recent reduction from 7.5mg/day 1 month ago)	HCQ 200mg/day
ARD control before vaccination	BVAS 3, Eos 0.20 (3 months prior)	N.A.	SLEDAI-2K: 0 points (12 months prior)	SLEDAI-2K: 2 points (3 months prior)	SLEDAI-2K: 0 points
Co-morbidities	HTN on lisinopril 10mg OM, HLD on simvastatin 20mg ON<br>Benign colonic polyps	Type II DM on metformin 500mg BD (HbA1c 6.7%), well-controlled asthma (no regular inhaler)	HTN on lisinopril of 5mg OM, recovered hepatitis B infection	HLD on diet control, vitamin D insufficiency	Nil
Doses of COVID-19 mRNA vaccine	#1 Moderna	#1 Pfizer	#1 Pfizer<br>#2 Pfizer	#1 Pfizer<br>#2 Pfizer	#1 Pfizer

Table S2: Features of active ARD and myocarditis post-COVID-19 vaccination and their management

	Patient 1	Patient 2	Patient 3	Patient 4	Patient 5*
Clinical summary					
Diagnosis and manifestations	EGPA flare – myocarditis, asthmatic exacerbation, polyarthralgia	Newly diagnosed active EGPA – myocarditis, adult-onset asthma, chronic rhinosinusitis, possible glomerulonephritis, hyper-eosinophilia, peripheral neuropathy, cutaneous vasculitis	SLE flare – myocarditis, thrombotic thrombocytopenic purpura, hypocomplementaemia	SLE flare – myocarditis, Evan's syndrome, fever, hypocomplementaemia	SLE flare – myopericarditis
ARD symptom onset after COVID-19 vaccination					
All	1 day after first dose	2 days after first dose	3 days after first dose	6 weeks after first dose<br>1 week after second dose	4 days after first dose
Cardio-respiratory	1 day after first dose	2 days after first dose	6-7 weeks after first dose<br>3-4 weeks after second dose	8 weeks after first dose<br>2 weeks after second dose	4 days after first dose
Myocarditis diagnosis					
Brighton CCD^<br>US CDC~	Definite<br>Confirmed	Definite<br>Confirmed	Definite<br>Probable	Definite<br>Probable	Probable<br>Probable
Investigations					
Haematology and biochemistry	WBC 11.4 Eos 0.88 Hb 14.5<br>Plt 215 Cr 71	WBC 12.0 Eos 5.39 Hb 11.5<br>Plt 143 Cr 76	WBC 7.6 Hb 4.9 Plt 16 Cr 206<br>Haptoglobin <30 LDH 2525<br>PBF: many fragmented cells seen	WBC 9.8 Hb 5.8 Plt 7 Cr 43<br>PBF: giant platelet some macrocytes, no schistocytes	Not reported
Peak Troponin I level	1236	1046	1066	48	159 Hs-trop (mg/L)

Inflammatory markers	CRP 7.5 ESR 25	CRP 31.4 ESR 112	CRP 12.8 ESR 112	CRP 7.7 ESR not done	CRP 68 ESR 92
Urinalysis	RBC 7 WBC 33 No protein on urine dipstick	RBC 113 (10% dysmorphic) WBC 1 24H UTP: negative	RBC 21 WBC 5 24H UTP: 1.18g/day	RBC 5 WBC 2 No protein on urine dipstick	Not reported
Serology	ANCA negative Anti-PR3 10 ; Anti-MPO <2	ANCA negative Anti-PR3 <2 ; Anti-MPO < 2	Anti-dsDNA < 25	Anti-dsDNA – not performed	Not reported
Cardiac					
Electrocardiogram	Normal sinus rhythm. Old changes of Q waves and TWI in V2, TWI in V3, 4.	Sinus tachycardia 115 bpm, ST segment depression + TWI in V4-V6	Sinus tachycardia, mild ST segment elevation II, III leads, AVF (early repolarization changes)	LBBB (no previous ECG)	Normal sinus rhythm
Transthoracic echocardiogram	LVEF 45% RWMA in LAD territory	LVEF 55-60%, multiple masses up to 15mm in size in LV, attached to papillary muscles	Reduced LVEF 50%, global hypokinesia, mild circumferential pericardial effusion, moderate TR	Reduced ejection fraction 50%, global hypokinesia	Pericardial effusion 0.3cm
Cardiac MRI	Marked focal edema, myocardial hyperaemia and non-ischemic pattern of delayed myocardial enhancement at the apical, anteroseptal and basal anterior myocardium	Non-specific myocardial edema and filling defects attributed to small thrombi in LV, attached to papillary muscles	Not done	Not done	Not done
Management					
Induction	Pulse MP 3g total IV CYC at 0.6g/m² BSA x 3 cycles	Pulse MP 1.5g total PO CYC ~0.76mg/kg/day	Pulse IV MP 3g total Rituximab 375mg/m² BSA weekly x 4 doses PLEX 22 sessions IV CYC at 0.5g/m2 BSA	IV MP 1.5g total Rituximab 1g x 2 doses Intravenous immunoglobulin 2g/kg body weight	PO prednisolone 25mg for 1 week then weaned to 5mg over subsequent 2 weeks.

Maintenance	AZA 50mg OM (~0.83mg/kg/day)	AZA 25mg OM ~0.35mg/kg/day after 8 months of CYC	MMF 1g/day	AZA 150mg OM ~2mg/kg/day	Not reported
Outcomes	Trop normalized Repeat TTE in 3m: LVEF of 60% with no RWMA	Trop normalized: Repeat TTE in 3w: resolution of multiple thrombi	Trop normalized Planned for repeat TTE 4m later.	Trop normalized Repeat TTE in 3m: improvement in LVEF to 55%	Symptoms improved. Repeat Trop and TTE not reported

^According to Brighton Collaboration Case Definition. Tejtela SKS, Munozb FM, Al-Ammouric I, et al. Myocarditis and pericarditis: case definition and guidelines for data collection, analysis, and presentation of immunization safety data2021.

~According to US CDC Case Definition. Gargano JW, Wallace M, Hadler SC, et al. Use of mRNA COVID-19 Vaccine After Reports of Myocarditis Among Vaccine Recipients: Update from the Advisory Committee on Immunization Practices - United States, June 2021. MMWR Morb Mortal Wkly Rep 2021 Jul 9;70(27):977-982. 2021.

* Patient 5 is a case described in Patel S, Wu E, Mundae M, Lim K. Myocarditis and pericarditis following mRNA vaccination in autoimmune inflammatory rheumatic disease patients: A single-center experience. Rheumatol Autoimmun. 2022;2(2):92-7DOI: 10.1002/rai2.12042.

Patient 1-4 is described in this case series

Note: ARD = autoimmune rheumatic disease. eGPA = eosinophilic granulomatosis polyangiitis. SLE = systemic lupus erythematosus. HTN = hypertension. HLD = hyperlipidemia. DM = Diabetes Mellitus. TTP = thrombotic thrombocytopenic purpura. APLS = antiphospholipid syndrome, APAs = anti phospholipid antibodies (anticardiolipin antibody, anti-Beta2 glycoprotein antibody, Lupus anticoagulant), bpm= beats per minute. ECG = electrocardiogram. TTE = transthoracic echocardiogram. TWI = T wave inversion. LV = left ventricular. EF = ejection fraction. RWMA = regional wall motion abnormalities. cMRI: cardiac magnetic resonance imaging of heart. TR = tricuspid regurgitation. UTP= urine total protein. ANCA= anti-neutrophil cytoplasmic antibody. SLEDAI-2K = systemic lupus erythematosus disease activity index -2000. BVAS = Birmingham Vasculitis Activity Score. IV = intravenous. MP = methylprednisolone. CYC = cyclophosphamide. AZA = azathioprine. HCQ = hydroxychloroquine. Pred = prednisolone. MMF = mycophenolate mofetil. PLEX = plasma exchange

Reference ranges:

White blood cell count (WBC): (4.0 – 9.6 x 10^9/L)

Hemoglobin (Hb): (13.6 – 16.6 g/dL)

Platelet (Plt): (150 – 360 x 10^9/L)

Eosinophil count (eos): (0.00-0.60 x 10^9/L)

Creatinine (Cr): (60-105umol/L)

Haptoglobin: (36-200mg/dL)

Lactate dehydrogenase (LDH): (270-550U/L)

uACR (0.0-2.5 mg/mmol).

erythrocyte sedimentation rate (ESR): (1-10mm/hr)

c-reactive protein (CRP): 7.5 (0.0 – 5.0mg/L).

Troponin I (Trop): (0-18ng/L)

Anti-double stranded DNA (anti-dsDNA): 0 – 25 IU/mL

Anti-proteinase-3 (anti-PR3): (0-20 RU/ml). Anti-myeloperoxidase (anti-MPO): (0-20 RU/ml)
